# Long-Term Neurological Sequelae and Disease Burden of Japanese Encephalitis in Gansu Province, China

**DOI:** 10.5334/aogh.3343

**Published:** 2021-10-21

**Authors:** Xuxia Wang, Li Su, Shougang Sun, Wenbiao Hu, Qiuling Mu, Xuefeng Liang, Na Jin, Tian Dai, Hui Li, Guihua Zhuang

**Affiliations:** 1 Department of Epidemiology and Biostatistics, School of Public Health, Xi’an Jiaotong University Health Science Center, Xi’an 710061, P.R. China; 2 Institute of Health Education, Gansu Provincial Center for Disease Control and Prevention, Lanzhou, 730000, China; 3 School of Public Health, Lanzhou University, Lanzhou 730000, P.R. China; 4 Department of Cardiology, Lanzhou University Second Hospital, Lanzhou 730000, P.R. China; 5 School of Public Health and Social Work, Queensland University of Technology, Kelvin Grove 4059, QLD, Australia; 6 Gansu health vocational college, Lanzhou 730000, P.R. China; 7 Institute of Immunization Programme, Gansu Provincial Center for Disease Control and Prevention, Lanzhou, 730000, China; 8 Institute of prevention and control of infectious diseases, Gansu Provincial Center for Disease Control and Prevention, Lanzhou, 730000, China

## Abstract

**Background and objects:**

The study aimed to evaluate the long-term neurological sequelae and the disease burden of JE in Gansu, China.

**Methods:**

JE patients were included as study population from 2005–2011 in Gansu, and a follow-up survey was conducted in 2007–2014. Pair-matched healthy individuals were selected as controls. All subjects underwent a neurological examination and intelligence quotient (IQ) and memory quotient (MQ) assessments. Then, the disability-adjusted life years (DALYs), and direct and indirect medical expenses were systematic assessed.

**Results:**

Forty-four point seven percent of the JE patients had objective neurological deficits, compared with 2.4% of controls. Subnormal intelligence was found in 21.2% of JE subjects, compared with 1.2% control who exhibited a mildly reduced IQ. Abnormal MQ scores were noted in 56.3% JE subjects, compared with only 12.7% controls. Prevalence of each sequelae caused by JE were significantly higher in adults than in younger subjects. Furthermore, median DALY lost due to JE was 9.2 per subject. Median economic cost of JE was approximately $2776.6 per subject and significantly higher in adults than in younger subjects.

**Findings and Conclusions:**

JE patients suffered from severe neurological sequelae and high disease burden, resulting in a significant downstream burden for both the patients (especially adults) and the healthcare system.

## Introduction

Japanese encephalitis (JE), affects a population of >3 billion people and it is prevalent in South Asia, Southeast Asia, East Asia, and the Pacific regions [1]. It is estimated that there are approximately 67,900 JE cases annually in 24 countries, with 50% occurring in China [2]. JE leads to significant morbidity and mortality, with a fatality rate of approximately 20–30%. Of survivors, 30–50% exhibit significant neurological sequelae such as impaired cognition, seizures, motor impairment, coordination dysfunction, and psychiatric disorders [2,3,4].

The GBD Study uses disability-adjusted life years (DALYs), which is one of the most widely used metrics for assessing the human burden of disease [4,5,6]. It has been estimated that 265,778 to 1,859,170 DALYs were lost to JE worldwide in 2005 [7,8]. Moreover, according to the World Health Organization’s (WHO) position paper, JE-associated DALYs were 709,000 in 2015 [9]. However, few studies have focused on disease burden due to JE in China.

In recent decades, the wide use of JE vaccines has caused the ongoing incidence of JE to decline considerably in some Asian countries [10,11,12]. However, the increasing trend of adult cases in many areas, including Korea and China, was gradually attracting attention [10,12,13,14]. Epidemiological and public-health efforts regarding the disease burden of JE have to date focused on elderly patients. Studies on JE sequelae require long periods of clinic follow-up. However, there have been few studies on the prevalence, incidence, clinical follow-up and assessment of disease burden imposed by JE in China, especially in western areas. Hence, we conducted this study to assess long-term sequelae in JE cases diagnosed from 2005 to 2011 in Gansu, China. We also explored the characteristics of its disease burden, including DALYs and economic burden with respect to age.

## Methods

### Study Subjects

All patient cases were identified from the China National Notifiable Disease Reporting System (NNDRS) from 2005 to 2011. The prefectures of Tianshui, Pingliang, and Longnan, which were home to approximately 95% of the JE cases in Gansu, were selected as project areas. Confirmation of research object was based on JE virus-specific immunoglobulin M (IgM) in cerebrospinal-fluid or serum samples, captured by Gansu Provincial Center for Disease Control and Prevention (GSCDC) using enzyme-linked immunosorbent assays (ELISA) (Beixi Kit; Shanghai B&C, China).

A pair-matched healthy control was selected for each patient in his/her resident sub-community by convenience sampling. Healthy individuals without histories of neurological disease were matched for age, gender, place of residence, and educational level with JE subjects. Age differences were limited to <1 year for toddlers (≤6 years), <2 years for children (7–14 years), and <3 years for adults (≥15 years) between the JE and control groups.

### Clinical history survey

The patient’s medical history data were reviewed by researchers. These data included general conditions, neurological symptoms, times of admission and discharge, outcomes, and direct hospitalization expenses.

### Follow-up

Our follow-up entailed retrospective reviews in 2007–2014. We scheduled appointments at the community clinics. Home interviews were made if the patients did not make the appointment. After efforts to trace the discharged patients, we located 85 subjects in 2007–2014.

We used the Liverpool Outcome Scale (LOS) to assess their outcomes and also made a careful investigation of treatment expenses, including direct/indirect expense in acute phase and treatment costs during rehabilitation.

Each single question score ranged from 2–5. The final LOS is the lowest number scored for any question single question. The score of 5, 4, 3 or 2, corresponds to an outcome classification of no, mild, moderate or severe functional impairment respectively. Death was scored as 1.

### Examinations

The same examination items, scale, and questionnaire were used for the follow-up groups of JE patients as well as the control cases. The questionnaire and intelligence and memory tests were completed by the GSCDC professionals, and the neurological examination was conducted by local clinicians.

Neurologists had conducted the neurological and cognitive examinations at hospital or CDC clinics at follow-up. The patients’ clinical symptoms included muscle strength, tone, and sensation; tendon reflexes; pathological reflexes; aphasia, cranial-nerve symptoms; mental state; and whether seizure or epilepsy had occurred were analyzed.

Intelligence testing had then been performed using the China Wechsler Young Children Scale of Intelligence (C-WYCSI; for children 4–6 years old), China Wechsler Intelligence Scale for Children (C-WISC; for children 7–15 years old), or Wechsler Adult Intelligence Scale Revised in China (WAISRC; for people ≥16 years old). This testing included verbal and performance scales. The intelligence quotient (IQ) had been generated by looking up table, based on verbal-IQ (VIQ) and performance IQ (PIQ) test results [15,16,17]. The Wechsler Memory Scale (WMS; Chinese version, types A and B) had been used to assess memory ability for subjects ≥7 years old. A comprehensive memory quotient (MQ) had been derived from the test results. Furthermore, the Children’s Adaptive Behavior Assessment Scale (CABAS) and the Mental Handicap Rating Scale for Adult (MHRSA) had been used to tested intellectual disabilities for subjects <4 years old and those unable to complete the intelligence test because of severe [18,19,20].

### Ethical considerations

The survey was approved by the Ethical Review Committee of the GSCDC (ethical approval number: GSCDC2013010). Written informed consent was obtained from JE subjects and healthy controls or from their proxies before the investigation.

### Data analysis

We used the McNemar’s test or Corrected chi-square (χ^2^) or Fisher’s exact test for the rates or percentage in different groups based on the characters of data, and student’s *t* test for means. Differences were considered to be statistically significant at *P* < 0.05.

We calculated DALYs lost due to confirmed JE for each patient from age, gender, and disability weight (DW), according to the guidelines in National Burden of Disease Studies: A Practical Guide (WHO, 2nd ed.). Moreover, we calculated life expectancies from the Western family model life table (USA) among different age groups [21,22,23]. We used DW of 0.616 (0.613–0.616) for episodes, 0.468 (0.402–0.484) for cognitive impairment and 0.379 (0.350–0.453) for neurological sequelae [22]. In addition, Severity of sequelae was varied with different individuals, we also confirmed DW value by the Liverpool Outcome Score (LOS). If the scale score is 2, the maximum value of DW is selected; if it is 3, the average value is selected; and if it is 4, the minimum value is selected. Most cases have multiple sequelae, total DW is maximum value for any single sequelae [24,25]. We assumed an acute phase duration of six months and lifelong disability. We assumed lifelong disability; we did not consider the YLL caused by sequelae when calculating DALYs.

We calculated the routine direct costs of JE cases using outpatient, hospital, transportation and health-food costs. We collected the relevant data from the hospital system or through interviews by CDC investigators. The investigators had consulted a list of hospital expenses, matching and confirming the follow-up information on hospitalization costs. We also calculated indirect costs using the human capital method, including income lost by both patients and caregivers due to missing paid work, as well as the cost of rehabilitation treatment. Therefore, total cost of JE was calculated by adding direct, indirect and rehabilitation treatment costs for each subject. All costs were discounted at 3% to constant 2011 Chinese currency (RMB) and then converted to US dollars using the 2011 RMB-US dollar exchange rate (6.4614 RMB to 1 US$).

## Results

### Research completion

From 2005 to 2011, a total of 486 JE cases were reported and 234 case specimens were tested. Among them, 156 (66.7%) were identified as positive by the presence of the JE virus IgM antibody, and 148 in the studied area. Socioeconomic and demographic profiles of the patients are provided in ***Table 1***.

**Table 1:** Demographic features of JE cases in Gansu, 2005–2011.


DEMOGRAPHIC CHARACTERISTICS	NO. (%) OF FOLLOW UP					NO. (%) OF LOST	NO. (%) OF TOTAL

	EXAMINATIONS	REFUSED	DIED				

			DIED OF JE	NOT DIED OF JE	TOTAL DIED		

**Ages(years)**							

0-	25(86.2)	2(6.9)	0(0.0)	0(0.0)	0(0.0)	2(6.9)	29(100.0)

6–	25(73.5)	5(14.7)	2(5.9)	1(2.9)	3(8.8)	1(3.0)	34(100.0)

15–	18(52.9)	7(20.6)	5(14.7)	0(0.0)	5(14.7)	4(11.8)	34(100.0)

45–76	17(33.3)	5(9.8)	18(35.3)	7(13.7)	25(49.0)	4(7.9)	51(100.0)

**Sex**							

F	39(52.7)	10(13.5)	16(21.6)	4(5.4)	20(27.0)	5(6.8)	74(100.0)

M	46(62.1)	9(12.2)	9(12.2)	4(5.4)	13(17.6)	6(8.1)	74(100.0)

**Career**							

Toddlers	25(80.6)	3(9.7)	0(0.0)	0(0.0)	0(0.0)	3(9.7)	31(100.0)

Students	28(75.7)	5(13.5)	2(5.4)	1(2.7)	3(8.1)	1(2.7)	37(100.0)

Farmers	25(39.7)	9(14.3)	20(31.8)	5(7.9)	25(39.7)	4(6.3)	63(100.0)

Others	7(41.2)	2(11.8)	3(17.6)	2(11.8)	5(29.4)	3(17.6)	17(100.0)

**Prefectures**							

Pingliang	25(56.8)	1(2.3)	12(27.3)	3(6.8)	15(34.1)	3(6.8)	44(100.0)

Tianshui	17(46.0)	7(18.9)	9(24.3)	1(2.7)	10(27.0)	3(8.1)	37(100.0)

Longnan	43(64.2)	11(16.4)	4(6.0)	4(6.0)	8(12.0)	5(7.4)	67(100.0)

**Year of discharge**							

2005–2006	66(65.3)	7(6.9)	18(17.8)	5(5.0)	23(22.8)	5(5.0)	101(100.0)

2007–2011	19(40.4)	12(25.5)	7(14.9)	3(6.4)	10(21.3)	6(12.8)	47(100.0)

**Total**	85(57.4)	19(12.9)	25(16.9)	8(5.4)	33(22.3)	11(7.4)	148(100.0)


**Figure 1:**
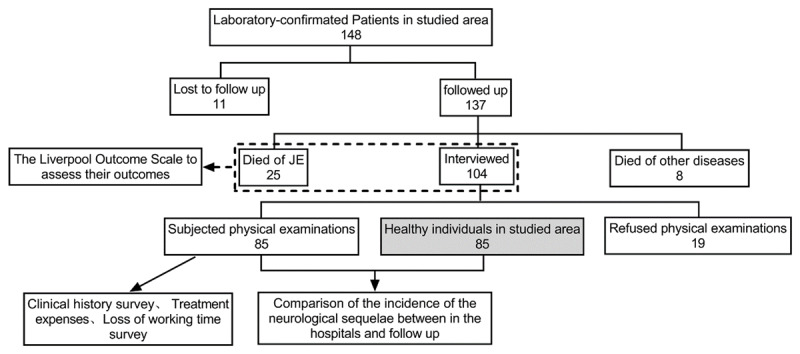
Summary findings and assessment for 148 JE patients followed up 8 years after hospital discharge.

Of the 148 patients, we followed up with 137 while 11 cases were lost. 24.1% (33/137) patients had died before we conducted this survey. 17.5% (24/137) died of JE, 0.7% (1/137) died of a JE sequela (epileptic seizure), 5.1% (7/137) died of other diseases and 0.7% (1/137) died in a traffic accident. Another 19 refused physical examinations. Combining case history information with a phone or household survey, we found that 8 of those 19 (42.1%) had one or more neurological or psychiatric sequelae, including delayed speech (n = 4), paralysis (n = 1), decreased vision (n = 4), and memory loss (n = 6). Finally, a total of 129 cases were investigated by LOS and 85 cases conducted intelligence and memory tests. The completion of the study is shown in ***Figure 1***.

### Neurological sequelae for JE and Healthy groups

Among the 85 JE patients, 74 (87.1%) had meningeal irritation, and 65 (76.5%) suffered loss of consciousness in the hospital, but none had these signs at follow-up (Data had not shown).

From hospitalization to 1–8 years post-discharge, 44.7% (38/85) of surviving JE patients still had at least one nervous-system abnormalities, compared with 88.2% (75/85) while still in the hospital (Data had not shown).

We also compared rates of neurological sequelae and psychiatric disorders in different age groups at the follow-up and found that adults (≥15 years) had a higher overall rate of neurological sequelae (*P <* 0.05). The percentage of symptoms in the control group was significantly lower than that in the JE group with at least one nervous system when followed up (*P <* 0.05; ***Table 2***).

**Table 2:** Comparisons of Neurological symptoms by age group.


AGE GROUP	NEUROLOGICAL SYMPTOMS (%)						MENTAL DISORDERS (%)	WITH ANY OF SYMPTOMS (%)

	CRANIAL NERVES SYMPTOMS	MOTOR NEURON SYMPTOMS	APHASIA	EPILEPSY OR TIC	SENSORY NEURON SYMPTOMS	WITH ANY OF NEUROLOGICAL SYMPTOMS		

**2–14 years (N = 50)**								

JE group	2(4.0)	11(22.0)	2(4.0)	6(12.0)	2(4.0)	17(34.0)	5(10.0)	17(34.0)

Healthy group	0(0.0)	0(0.0)	0(0.0)	0(0.0)	0(0.0)	0(0.0)	0(0.0)	0(0.0)

McNemar or Corrected χ^2^	0.5	25.0	0.5	4.2	0.5	15.1	3.2	15.1

*P* value	0.50	0.00	0.50	0.03	0.50	0.00	0.06	0.00

≥**15 years (N = 35)**								

JE group	2(5.7)	18(51.4)	5(14.3)	2(5.7)	21(60.0))	21(60.0)	9(25.7)	21(60.0)

Healthy group	0(0.0)	2(4.0)	0(0.0)	0(0.0)	0(0.0)	2(0.0)	0(0.0)	2(4.0)

McNemar or Corrected χ^2^	0.5	11.3	3.2	0.5	19.1	14.1	7.1	14.1

*P* value	0.50	0.00	0.06	0.50	0.00	0.00	0.00	0.00

**Total (N = 85)**								

JE group	4(4.7)	29(34.1)	7(8.2)	8(9.4)	23(27.1)	38(44.7)	14(16.5)	38(44.7)

Healthy group	0(0.0)	2(2.4)	0(0.0)	0(0.0)	0(0.0)	2(2.4)	0(0.0)	2(2.4)

McNemar or Corrected χ^2^	2.3	21.8	5.1	6.1	21.0	30.6	11.3	30.6

*P* value	0.13	0.00	0.02	0.02	0.00	0.00	0.00	0.00


### Intelligence and memory assessment for JE and Healthy groups

**Figure 2:**
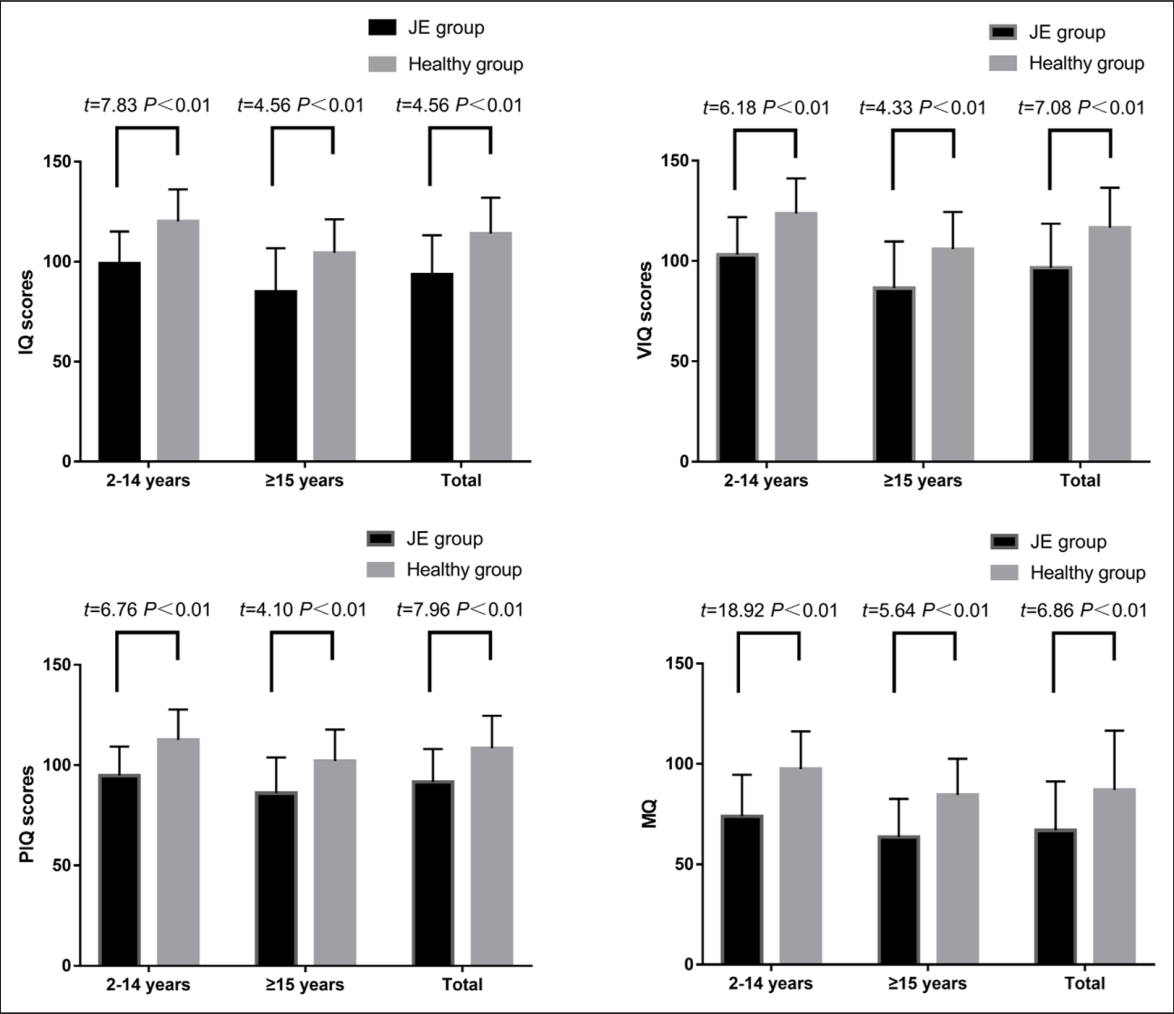
Quantitative analysis of results of intelligence and memory by age group.

In total, 74 pairs of JE patients and healthy controls underwent intelligence testing. Nine JE subjects could not participate in the testing because of severe psychiatric disabilities, and two were unsuitable for intelligence testing due to being <4 years old, so they underwent disabled-adult intelligence or child adaptability testing instead. Among the 74 pairs, 71 pairs older than seven years underwent memory testing. In the JE group, the average IQ, VIQ, PIQ, and MQ scores were significantly lower than in the control group (*P* < 0.01; ***Figure 2***). Consequently, incidence of low intelligence and memory was significantly higher in the JE group than those in the control group (*P* < 0.05; ***Table 3***). Moreover, prevalence of lower IQ, VIQ, and PIQ scores (<70) were significantly higher in adults (age ≥15 years old) than in children (<15 years old) with JE (*P* < 0.01). Poor memory was also higher in the adult group (*P* < 0.05).

**Table 3:** Comparisons of intelligence and memory by age group.


AGE GROUP	INTELLIGENCE EXAMINATION (%)			DEMENTIA	TOTAL LOWER INTELLIGENCE (%)	LOWER MEMORY
	
	IQ < 70	VIQ < 70	PIQ < 70			MQ < 70(%)

**2–14 years**	N = 45	N = 45	N = 45	N = 5	N = 50	N = 42

JE group	1(2.2)	0(0.0)	1(2.2)	4(80.0)	5(10.0)	19(45.2)

Healthy group	0(0.0)	0(0.0)	0(0.0)	0(0.0)	0(0.0)	2(4.8)

McNemar or Corrected χ^2^	0.0	N/A	0.0	2.3	3.2	15.1

*P* value	1.00	N/A	1.00	0.13	0.06	0.00

≥**15 years**	N = 29	N = 29	N = 29	N = 6	N = 35	N = 29

JE group	7(24.1)	7(24.1)	8(27.6)	6(100.0)	13(37.1)	21(72.4)

Healthy group	1(3.4)	1(3.4)	1(3.4)	0(0.0)	1(2.9)	7(24.1)

McNemar or Corrected χ^2^	4.2	4.2	5.1	4.2	10.1	10.6

*P* value	0.03	0.03	0.02	0.03	0.00	0.00

**Total**	N = 74	N = 74	N = 74	N = 11	N = 85	N = 71

JE group	8(10.8)	7(9.5)	9(12.2)	10(90.9)	18(21.2)	40(56.3)

Healthy group	1(1.4)	1(1.4)	1(1.4)	0(0.0)	1(1.2)	9(12.7)

McNemar or Corrected χ^2^	5.1	4.2	6.1	8.1	15.1	27.3

*P* value	0.02	0.03	0.01	0.00	0.00	0.00


### Functional impairment following JE

**Figure 3:**
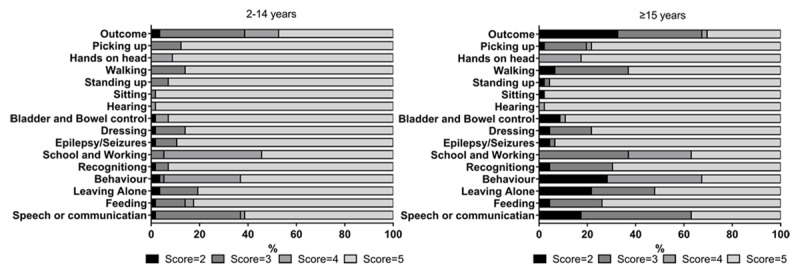
Comparisons of influence of functional impairment by age group for surviving cases.

Sixty-two (60.2%) of the 103 cases followed-up had functional impairment (LOS 2–4). Among the cases, there were no significant differences between the adults (≥15 years) than Child (2–14 years old) cases in impairment (69.6% [32/46] versus 52.6% [30/57]; *P* > 0.05). Severe impairment (LOS 2) was more common in adults than Child (32.6% [15/46] versus 3.5% [2/57]; *P* > 0.05). Influence of functional impairment is shown in ***Figure 3***.

### Median DALYs lost per patient

Median DALYs lost due to JE were 9.2 per patient, consisting of 11.5 YLLs per death and 7.2 YLDs per survivor (***Table 4***). Per patient, the burden in females was 9.3, in males 8.7. Age-specific median DALYs lost were 13.5 and 8.7 per patient for those ages <15 and ≥15 years old, respectively.

**Table 4:** Median DALY (P25, P75) lost per patient.


GENDER GROUP	DEATH		SURVIVAL		TOTAL	

	N	YLL	N	YLD	N	DALY

**Male**						

2–14 years	1	37.2(37.2, 37.2)	30	6.7(0.0, 15.4)	31	13.5(0.0, 16.5)

≥15 years	8	11.3(8.5, 24.5)	27	6.5(0.0, 11.1)	35	8.6(0.0, 11.5)

Sub-total	9	11.5(8.6, 28.4)	57	6.5(0.0, 14.2)	66	8.7(0.0, 14.2)

**Female**						

2–14 years	1	37.6(37.6, 37.6)	27	13.1(0.0, 16.7)	28	13.4(0.0, 17.0)

≥15 years	15	11.0(9.3, 19.3)	20	6.5(0.0, 9.8)	35	9.2(5.5, 12.2)

Sub-total	16	11.3(9.3, 21.9)	47	7.7(0.0, 16.3)	63	9.3(0.0, 16.7)

**Total**						

**2–14 years**	2	37.4(37.2, 37.6)	57	13.1(0.0, 16.6)	59	13.5(0.0, 16.7)

≥**15 years**	23	11.2(8.8, 19.3)	47	6.5(0.0, 10.8)	70	8.7 (4.9, 11.8)

**Sub-total**	25	11.5(9.0, 22.8)	104	7.2(0.0, 14.6)	129	9.2(0.0, 15.1)


### Direct and indirect expense

Median total direct expense was $1,644.0 ($913.1 to $2,883.3) in the acute phase. Direct cost in the adult group (≥15 years old) was $2,029.0, which was higher than in the younger group (<15 years old; $1137.4; *P* = 0.04) (***Table 5***). Direct hospital expense was also higher in the adult group ($1,779.8) than that in the younger group ($888.0; *P <* 0.05). Median total indirect expense was $371.4 ($19.3 to $832.8). Total indirect expense did not differ between the adult group ($533.9) and the younger group ($346.5; *P* > 0.05).

**Table 5:** Comparison of direct and indirect medical expenses in the same age group ($).


EXPENSE	AGE		TOTAL

	2–14 YEARS	≥15 YEARS	

**Direct expense in acute phase (N = 60)**	1137.4 (827.4, 2600.6)	2029.0 (1264.1, 4239.2)	1644.0 (913.1, 2883.3)

Outpatient cost before hospitalization (N = 60)	35.6 (1.7, 57.0)	26.7 (0.0, 72.1)	26.7 (0.4, 56.4)

Hospital costs (n = 85)	888.0 (534.0, 1748.8)	1779.8 (1022.7, 3559.6)	1263.7 (637.7, 2435.2)

Transportation fee (n = 85)	51.4 (27.0, 138.6)	53.4 (7.1, 251.7)	53.4 (18.9, 179.5)

Health-food costs (n = 60)	109.6 (31.9, 253.0)	154.8 (0.0, 178.0)	144.2 (8.0, 178.0)

**Indirect expense in acute phase (n = 60)**	346.5 (77.1, 800.9)	533.9 (0.0, 913.9)	371.4 (19.3, 832.8)

**Treatment costs during rehabilitation (n = 60)**	145.9 (87.7, 478.2)	889.9 (430.9, 1423.8)	246.2 (102.6, 978.9)

**Total costs (n = 60)**	1908.1 (1147.7, 4236.1)	3986.8 (2357.1, 5988.2)	2776.6 (1363.5, 5598.9)


Median cost (P25, P75).

## Discussion

JE infection was associated with a high frequency of long-term disability with neurological and developmental impairment for both young children and adults, 18.2% follow-up subjects died of JE and its sequelae, and JE mortality in adults was almost four times higher than that in children. The incidence of neurological abnormalities was 37% and 26% in pediatric and adult JE patients after one year, respectively [3,25]. 63% of JE subjects had neurological sequelae at discharge, and 56% of these had neurological sequelae two years post-discharge [26]. In this study, we found that 87.1% of JE subjects had neurological abnormalities during hospitalization, and 44.7% of these still had neurological abnormalities 1–8 years post-discharge. The rate of such sequelae in JE survivors 1–8 years post-discharge was similar to that found in a previous study in Gansu, which is 43.6% 1–2 years post-onset [27]. Motor nerve symptoms are the main neurological sequelae in JE patients, and a larger proportion of patients had residual motor nerve symptoms 1–8 years later compared with controls. These sequelae included paralysis, hemiplegia, limb movement disorder, reduced muscle strength, reduced tendon reflexes, involuntary movements, and increased muscle tone.

Some studies reported that young children (<10 years old) are more likely to have residual neurological impairments than adults [27,28,29]. Yin et al. have suggested that rates with any neurological sequelae and psychiatric disorders among adult JE patients are similar to those of the groups of 2–5 and 6–14 year-olds [27]. In our study, rates of neurological sequelae were higher in adults (≥15 years old) than those in children (2–14 years old) with JE. Differences between our results and Yin’s may be due to inconsistent age groups or different years surveyed.

Whether in adults or children, the prevalence of lower IQ in JE patients was higher than that in the unaffected population. This difference is reflected not only in the function of the left hemisphere of the brain, which is measured by VIQ, but also in that of the right hemisphere as measured by PIQ, especially in adults with JE. Prevalence of lower IQ (<70) in confirmed JE cases was 2.2% in children 2–14 years old and 24.1% in adults ≥15 years old. Previous studies have reported that prevalence of lower IQ is 14.1%–32.0% in JE patients [3,27]. Moreover, MQ in the JE group was lower than in the control group in this study. Nearly 60% of subjects in the JE group had memory problems, but only 12.7% of healthy controls had a low memory score, clearly indicating that memory problems might occur before intellectual ones in JE. Because the Wechsler intelligence scale cannot be used in cases of serious psychiatric impairment, our study evaluated the adaptability in children and intellectual disabilities in adults who were unable to complete the intelligence test, providing a more complete evaluation of the intelligence of all subjects and clarifying JE’s effect on intelligence.

In the present study, the age-group-specific DALY lost values were derived from DALY lost values for individual patients. The DALY lost for each patient was generated directly from the patient’s age, gender, and fine-grained disability level, which determines reductions in life expectancy and optimum health during their residual life. Therefore, our projection is likely to be the best estimate of the DALY lost to JE in the Gansu population, while maintaining comparability to the WHO’s framework. The median DALY burden was 9.2 per patient, 8.7 for males and 9.3 for females, and 13.5 for children and 8.7 for adults. These results clearly indicate that JE imposes a great disease burden in Gansu.

It is difficult to recover from the sequelae of JE, which include neurological symptoms and psychiatric impairment; thus, the presence of sequelae increases the economic burden of JE [30]. However, the relative importance of JE in adults has yet to be addressed as a public-health priority because data on JE disease burden are usually lacking. In our study, the median total expense for JE patients was $2776.6, including $1644.0 for direct expenses and $371.4 for indirect expenses in the acute phase, and $246.2 for treatment costs during rehabilitation; all the expenses resulted from JE increased with the patient’s age. Our study demonstrates a great financial burden caused by JE for both children and, especially, adults.

Based on the findings, JE patients were left with neurological and neuropsychiatric deficits even 7–8 years post-discharge, suggesting a high frequency of long-term neurological sequelae and a profound disease burden for both children and, especially, adults. There are evidences that childhood cases have been greatly reduced by immunization with vaccines, and then the age distribution of cases has shifted toward adults in some areas of Mainland and Taiwan of China, especially in the elderly [31,32,33,34]. Therefore, we suggest that JE surveillance and immunization programs should be strengthened with an aim to reduce JE in adults.

Except for the partial loss of patients, another limitation to this study is that the cognitive-function test was judged by the information provided by the spouse or parent and the interview with subjects, which is a subjective assessment method that is inclined to approach normal levels in an effort to spare the patient embarrassment. Therefore, several patients’ cognitive-function may be overestimated.
